# Remarks on the Nature and Treatment of Tuberculosis*Read before the Michigan State Medical Society, Flint, 1892.

**Published:** 1892-07

**Authors:** E. L. Shurly

**Affiliations:** Detroit, Mich.


### REMARKS ON THE NATURE AND TREATMENT OF TUBERCULOSIS.*

BY E. L. SHURLY, M. D., DETROIT, MICH.

*Mr. Chairman, Ladies and Gentlemen—*

I regret very much that I have not a written paper for this occasion, as I intended to have. I shall therefore have to ask your indulgence for the deranged manner in which the subject will be presented.

As I understand the topic I am expected to speak of, it is "The Nature and Treatment of Tuberculosis." Of course, such a subject is of too wide a range, and will take up too much of your time for this occasion, and I shall therefore condense very much what I want to say, leaving out almost entirely any remarks upon pathology, which is very important and interesting.

Allow me to say first, that I think a great deal of confusion arising at the present time over the subject of tuberculosis, comes from lack of recognition of the several different states of the disease met with. There is no doubt but that it will be a long time before all the problems connected with the subject can be solved, and that many theories will have to be evolved. We are in the habit of sneering a little bit (if we be practical people) at the theories. After a l it is theory which must lead to the practical solution of all such questions.

Now, the prevalent theory is the bacilliary one; that is, that the bacillus tuberculosis is the cause of all these several different states known as tubercular disease. We heard this morning a very excellent paper on tuberculosis of the joints, by Prof. Nancrede, a very philosophical and instructive paper, and you will remember that the author spoke of the bacilliary action in the production of tuberculosis of the joints, although he did not insist upon it as inevitable. There are other surgeons who believe that the bacillus tuberculosis has nothing whatever to do with such diseases of the joints. There are still others who do not believe that many of these

---

*Read before the Michigan State Medical Society, Flint, 1892.

cases are tuberculosis. Of late years several bacteriologists have discovered differences in the form and behavior of these bacilli, in their morphology, and I think the present tendency of opinion is towards the view that there are really several kinds of tubercle bacilli. In a very able paper, published in the *Revue de Medicine*, last October, Dr. J. Courmont, who spent a great deal of time in the investigation, shows that he had isolated three or four different families of tubercle bacilli. Now, is it not possible that all of these different families or varieties of bacilli may have different physiological characteristics so to speak. While the morphology may not differ one from the other very much, physiology or life of the microbe may.

Then again there springs up in an etiological sense, the great question of the ferments accompanying them. The universal decision that tubercular affections are due to operations of bacilli is based largely upon elaborate and convincing experiments which are about as follows: The microbe is cultivated in a certain organic medium, then transferred from that to an animal; in the animal in turn is developed a tuberculosis, which again can be transmitted by inoculation to another animal, and so on; the disease in each having the same pathological features. Now, while this seems so plain, we lose sight of the fact (which the chemist tells us) that there is present a ferment. What is the agency of the ferment? Recently, Prof. Tamman has brought forward some views which are quite new and seem well proven, viz: that there are in many organic materials certain unformed ferments, which are in given instances, as it were, in a state of equilibriumone with the other; that the least thing may disturb this equilibrium and cause the formation of active products; even a little acid to one or a little water to another will materially set the action going or change the result. This is a very important point to consider in connection with inoculating experiments, so that when we come to think of transferring this culture or that to an animal, we do not know how much or how little of this ferment we transfer also. I do not believe anybody can stand up and with absolute certainty say that he has transferred nothing but the bacillus. He must have transferred with the microbe some unformed or active ferment, either the product of the bacillus or extraneous to its life.

And therefore we do not know how much or how little the disease process depends upon the ferment which is transferred with the microbe or the microbe itself. Hence, I believe that in the present state of our knowledge, it is unsettled whether the microbe or bacillus is the only cause of the diseased condition generally classed as tuberculosis. I also believe, and I think the drift of opinion is in this direction, that there are distinct clinical and pathological differences in these cases. For instance, I do not think from a clinical standpoint that so-called tuberculous joints, so-called tubercular pulmonary disease, leprosy, lupus and tuberculosis of the peritoneum are identical diseases. I do not believe they are one and the same disease, although they may be closely related in some particulars. That brings us then to the practical consideration of the classification of such diseases. Every practitioner knows that there are certain forms or cases of disease, which he is unable to classify exactly by physical signs or by clinical history, and that there are certain differences clinically, no matter what identity laboratory examination may show regarding them. Some of these variations may be accounted for partly by the individual tendency to peculiarities, individual idiosyncrasies, but such consideration is not sufficient to account for the regular course of special cases. For instance, we have a case where there is no hemoptysis, where the cough comes on gradually but is a first symptom, while pyrexia and adynamia come on later and progressively, and after a while expectoration, etc. Upon physical examination of the chest we find that there is gradual consolidation taking place in the upper part of one lung, and perhaps some lesion in the other lung, all of which progresses, the patient dies, and if an autopsy is made, there will be shown the ordinary morbid anatomy of a case of pulmonary tuberculosis. The course may cover a period of several months, a year or more. Yet another form will cover a period of several years. Still another form, the mechanical, such as miners and grinders are afflicted with, where a great many years are consumed in the course of the disease. And again, another very decided form, where the course is very rapid but differing from acute miliary tuberculosis in this respect, that the lesion initially and all the time is confined to the lung; after a little time the whole pulmonary tissue seems to liquefy. Such cases are typical. You will all recognize them. We meet with them frequently, but they are not one and the same thing, and moreover it does not change the result whether the family history be bad or good always, but if the domestic history has been good or bad, it may modify the course of the disease. These cases then are so frequently typical that we must recognize them at least as varieties of pulmonary disease; in all of these examples mentioned, the lungs have been the seat of the principal lesion, therefore, they must be cases of phthisis. Now, we believe that all of such cases of so-called phthisis pulmonalis are essentially inflammatory; that the tubercular process is a secondary one. That the majority are ordinary catarrhal pneumonias, and whatever of tuberculosis comes, is secondary. This view, however, does not militate against the prevalent theory of the bacilliary origin of tuberculosis or phthisis pulmonalis, inasmuch as it is said by every one that there must be a nidus for the germs to grow upon. Why they do not always occur and develop in all cases of catarrhal inflammation of the air passages is a mystery. However, we know as a fact they do not. Allow me for a moment by way of diversion to call your attention to laryngeal phthisis, one of the most horrible diseases that can affect the human being. This disease as you know is very obstinate. It appears in two general forms, and has peculiar features. There are very few cases of laryngeal phthisis which are secondary to pulmonary tuberculosis. It is often coincident to pulmonary disease, it is true, but it is not often secondary. Yet what a large number of cases of pulmonary phthisis occur without any laryngeal complication whatever, without laryngeal phthisis. Is it not strange?

In chronic laryngitis, even with erosion, no pulmonary phthisis, no tuberculosis necessarily follows. Why should this be, if the bacilli are constantly travelling this track, and only waiting for a little opening good ground? Hence there must be other etiological factors to be taken into consideration in any description of the nature of this disease, called pulmonary phthisis. To sum up, I should be inclined to think that the nature of pulmonary tuberculosis or pulmonary phthisis was essentially inflammatory in the beginning, whatever subsequently may follow. This view does not seem unreasonable in the light of clinical history, but I admit it may seem unreasonable in the light of laboratory experiment as conducted at the present day. Deduction from these, however, are undergoing great change. If it be true that common phthisis pulmonalis be an inflammatory affection, it gives us some encouragement toward endeavors to mitigate its inevitable course and result. On the other hand, if it be always specific, dependent upon the invasion and operation of bacilli alone, then there is no hope. Regarding treatment, which has been generally unsatisfactory, we find many difficulties.

At the outset in almost all cases, one of the most troublesome symptoms we meet with is anorexia, which precludes the establishment of any sort of nutrition for the benefit of the patient, and which constitutes one great drawback to all treatment. Perhaps I am a little ahead of my story, for I ought to specify that we have three classes of cases to deal with: acute, sub-acute, and chronic. That is excluding what is called miliary or general tuberculosis.

But anorexia is common to all, and one of the first difficulties to be overcome and thought of. Those of us who have had large experience with these diseases, become very much discouraged in attempting to keep up the nutrition of the patient on account of this symptom, especially if we are obliged also to administer by the stomach all sorts of medicines and oils, etc. But this has been the ordinary mode of treatment-the applications of medicines in the usual way, by the stomach. And we know how very few patients will tolerate it for any great length of time without greater increase of anorexia; this is especially true of the cough balsams and syrups and oils. Therefore, it seems very desirable to adopt any method of which medication may be useful outside of stomachal. Now we have two things before us in this direction, one is climatic treatment and the other is by means of inhalation or hypodermic medication, or both. Climatic is so efficacious and well known that I need not speak of it here. I would remind you, however, as a proof that the bacillus is not the only cause of the diseases in question, that this microbe will operate just as well in Colorado and California as in Michigan, notwithstanding the claims of enthusiasts that the atmosphere of the former States is a septic, and if not indigenous there they are taken there, and probably flourish, since cases of phthisis pulmonalis or tuberculosis originate there and run the same course just as in Michigan. But outside of this we do know that a favorable climate is one of the most useful factors in treatment, for by putting the patient in a position to get plenty of fresh air, oxygen (as necessary as other nutrition) by putting him in the way of receiving plenty of heat and light, and the benefit of the actinic rays of the sun together with dry atmosphere, he is undoubtedly placed in a position of great advantage.

Regarding medication, we consider of first importance the hypodermic method because the medicine is introduced into the system in its pure state, undergoing no change by contact with the secretions of the stomach or intestines, besides whatever good it does in a selective way, the system may get the full benefit. This we believe is the preferable way, whether the disease be much localized in the lung or not. Notwithstanding particular localization, there is always spontaneously generated from this source a certain amount of septic poison, so that cases of phthisis pulmonalis are essentially forms of septic cæmia, a general septicæmia of some sort induced by the circulation of some toxic material which poisons the tissues generally. This seems perfectly rational, and ought to be very generally accepted. Now, if we can introduce something into the economy that will neutralize the septic material, of course we take a step forward. It is claimed by many that nothing but a colloid substance—organic—can be expected to act permanently in this way to neutralize an animal poison. We have not time to go into this question, which together with a great many other such questions may be relegated to the chemist, who in the near future must settle many mooted points of pathology. I will only say that this is certainly not borne out in practice. For we may introduce an alkaloid into the general system with as much hope of its producing a permanent, prompt and selective effect as though it were colloid; it is not necessary, then, that it be an animal poison. However, while we can produce such effects from an alkaloid just as well as a colloid, the effect is not as lasting because there are no secondary or multiplication effects excepting upon the nervous system, so that the injections must be repeated.

Therefore, I still believe that is the proper and rational method of systematic treatment. We prefer iodine and chloride of gold and sodium, but perhaps the use of iodine and chloride of gold and sodium may not be the best chemicals. There may be found some others that will answer the purpose better; undoubtedly there will be. But these two chemicals have done much good in pulmonary phthisis of the earlier stages. But of course they are not capable of controlling the septicæmia which occurs in a large class of cases. Take, for instance, a case showing evidence of tuberculosis affecting the mesenteric glands as well as the lungs, it is very difficult to put enough of any chemical into the system without getting a toxic effect, to neutralize it. Besides this method, we may be able to modify the source of poisoning by proper inhalations—antiseptic inhalations of some sort—when there are patches of caseation that are accessible to the bronchial tubes, by the use of some gas which can reach that part. In this way a cutting off of the source of septic material may be obtained, and for that reason different gases have been used from time to time. Chlorine gas has been used at different times, but abandoned because irrespirable. However, it has been found practical for inhalation when properly used, mixed with salt spray, and the cases properly selected for it. We may use also the antiseptic balsams for the same purpose. These not only have a local effect in quieting the immediate symptoms known as cough and so on, but certainly have the ulterior effect of cutting off the supply of septic material. So that, so far as the therapeutic indications are concerned, there seems to be two—one to limit the extension of the disease, and the other to neutralize the septic material that is being generated from hour to hour in the course of the disease.

One thing that for many years has given me great hope that we might achieve something in the treatment of phthisis pulmonalis is, that a great many of the local changes, the pathological changes, which take place in the lung are really conservative. They are nature's efforts to limit the disease and to throw off the *materies morbi*, whatever it may be.

I don't think I will take up your time in giving the history of cases *in extenso.* I would simply like to mention two or three illustrating the remarks. For instance, one case that I have only a synopsis of, is as follows:

Mr. S., age 37, farmer, German; father and grandfather died of phthisis. One year before entering hospital he had bronchitis, and for six weeks was quite sick with it; but finally grew better. He has had cough ever since, with expectoration increasing until it became abundant and heavy. When he was admitted to the hospital his pulse was small and rapid, 120, temperature, 101.5°. There were tubercle bacilli in sputum in great quantities. He was put upon a treatment of hypodermic injections of iodine, commencing with one-sixth grain, which was rapidly increased to a grain and a grain and a half, together with chlorine inhalations.

The physical signs showed the upper half of the right lung more or less consolidated, as shown by bronchial respiration, increased fremitus, bronchophony, and dullness on percussion. This condition extended down to about the fifth rib in front and behind. The respiratory murmur on the other side was exaggerated. Now this is a type of case commonly met with—a case of phthisis beyond incipiency. This man also had a bad family history. The temperature and pulse range gradually, but quite rapidly, diminished under the treatment, so that at the end of the second week the evening temperature did not go beyond 99.6° and the pulse subsided to 90; at the end of the fourth week the evening temperature ranged at 90°, and the pulse at 84, and there were no other exacerbations through the day. There had been a return of appetite, and all the physical functions were being performed very well.

This man was in the hospital about eight weeks; he went home last week. Now, you can't say that the man is well. He may have a relapse, and some more inflammation of the lungs, and he may have tuberculosis yet, but I do not believe that his was a case of tuberculosis. I believe the diagnosis between phthisis pulmonalis and tuberculosis must be made by some other method than those in vogue now. We shall have to take into consideration other characteristics of the sputum besides bacilli. We have already found that in some cases there are certain precipitates from the sputum which will kill animals directly, while the precipitates obtained with the same re-agents from other cases will not kill animals. Whether this arises from some deflections by chemical manipulation, or whether there is some peculiar toxine we cannot say. As it occurs so uniformly, we believe that there must be something in it of importance before we can arrive at the proper diagnosis, prognosis and classification of these diseases. In all probability we shall have to call in the aid of a chemist to examine chemically the expectoration, and trust as now to the staining of bacilli.

Another case I will relate, where a man was under treatment three years ago, who seemed apparently to recover, although throughout the course of his disease he had some looseness of the bowels, showing some intestinal disturbance; he improved and left the hospital after two months. He returned six weeks ago very much worse. He seems to be getting better again, although there is diarrhœa and tenderness of the abdomen. Now, the probability is he will not recover under the treatment. He had also a bad family history. Now, whether this case is of a different nature, really a tuberculosis or a general glandular degeneration or not, we cannot say. I could relate another case, if there were time, bearing on the paper of the gentleman who preceded me—a case we now have in the hospital of general tuberculosis, probably showing no lesions in the lungs. The physical signs show no consolidation. Respiration is simply rapid from pyrexia. We are unable to make the diagnosis exactly. We simply know it is not typhoid fever. The man is rapidly going to his death; there seems to be no lesions discoverable by auscultation in the chest, which will at all account for the patient's condition. Neither are the abdominal lesions sufficient to account for the great pyrexia and adyramia. It seems to be a systematic infection.

A case of this sort should be classified not as a case of phthisis pulmonalis, as is generally done. It is undoubtedly a case of tuberculosis. Therefore, I believe, that adopting the idea of phthisis pulmonalis being essentially a local inflammatory affection, while general tuberculosis is a primary general affection, will very much aid us in getting the true relative value upon therapeutic measures and plans, and enable us to classify these diseases with more propriety than heretofore.

---

We would direct the attention of our readers to the advertisement of Henry W. Wampole & Co., whose elegant preparations of cod liver oil, compound syrup of hypophosphite, and tasteless cascara bark, have of late received such an amount of justly merited praise. This altogether reliable house is to be congratulated upon the success they have acheived, and to our readers and the profession at large we cheerfully commend them and their most excellent preparations.

